# Bovine Lactoferrin Pre-Treatment Induces Intracellular Killing of AIEC LF82 and Reduces Bacteria-Induced DNA Damage in Differentiated Human Enterocytes

**DOI:** 10.3390/ijms20225666

**Published:** 2019-11-12

**Authors:** Maria Stefania Lepanto, Luigi Rosa, Antimo Cutone, Mellani Jinnett Scotti, Antonietta Lucia Conte, Massimiliano Marazzato, Carlo Zagaglia, Catia Longhi, Francesca Berlutti, Giovanni Musci, Piera Valenti, Maria Pia Conte

**Affiliations:** 1 Department of Public Health and Infectious Diseases, University of Rome La Sapienza, 00185 Rome, Italy; mariastefania.lepanto@uniroma1.it (M.S.L.); luigi.rosa@uniroma1.it (L.R.); mellanijinnett.scotti@uniroma1.it (M.J.S.); antoniettalucia.conte@uniroma1.it (A.L.C.); massimiliano.marazzato@uniroma1.it (M.M.); carlo.zagaglia@uniroma1.it (C.Z.); catia.longhi@uniroma1.it (C.L.); francesca.berlutti@uniroma1.it (F.B.); piera.valenti@uniroma1.it (P.V.); 2 Department of Biosciences and Territory, University of Molise, 86090 Pesche, Italy; antimo.cutone@unimol.it (A.C.); giovanni.musci@unimol.it (G.M.)

**Keywords:** bovine lactoferrin, AIEC LF82 strain, *Escherichia coli*, CEACAM-6, histone γ-H2A.X, Comet assay, protection from DNA damage

## Abstract

LF82, a prototype of adherent-invasive *E. coli* (AIEC), is able to adhere to, invade, survive and replicate into intestinal epithelial cells. LF82 is able to enhance either its adhesion and invasion by up-regulating carcinoembryonic antigen-related cell adhesion molecule 6 (CEACAM-6), the main cell surface molecule for bacterial adhesion, and its intracellular survival by inducing host DNA damage, thus blocking the cellular cycle. Lactoferrin (Lf) is a multifunctional cationic glycoprotein of natural immunity, exerting an anti-invasive activity against LF82 when added to Caco-2 cells at the moment of infection. Here, the infection of 12 h Lf pre-treated Caco-2 cells was carried out at a time of 0 or 3 or 10 h after Lf removal from culture medium. The effect of Lf pre-treatment on LF82 invasiveness, survival, cell DNA damage, CEACAM-6 expression, apoptosis induction, as well as on Lf subcellular localization, has been evaluated. Lf, even if removed from culture medium, reduced LF82 invasion and survival as well as bacteria-induced DNA damage in Caco-2 cells independently from induction of apoptosis, modulation of CEACAM-6 expression and Lf sub-cellular localization. At our knowledge, this is the first study showing that the sole Lf pre-treatment can activate protective intracellular pathways, reducing LF82 invasiveness, intracellular survival and cell–DNA damages.

## 1. Introduction

The bacterial ability to invade host cells is a pathogenic mechanism which can have serious effects on the establishment, persistence, severity and propagation of infections. The intracellular environment represents a privileged niche for bacterial survival and growth. Several bacterial genera are defined intracellular facultative pathogens, and among these, *Escherichia coli* LF82 is not an exception.

*Escherichia coli* LF82 strain, prototype of adherent-invasive *E. coli* (AIEC) able to adhere to and invade intestinal epithelial cells (IECs) [1,2], colonizes at high-extent ileal mucosa in Crohn’s disease (CD) patients [3,4,5,6].

Its adhesion is mediated by type 1 pili (FimH) via interaction with carcinoembryonic antigen-related cell adhesion molecule 6 (CEACAM-6) that is over-expressed at the apical surface of IECs in CD patients [7]. Additionally, AIEC LF82 strain further raises CEACAM-6 expression on the surface of IECs, facilitating its adhesion [2,7,8].

LF82 survival and replication have been observed in the cytoplasm of epithelial cells [9,10] as well as in macrophages, where it does not induce apoptosis [11,12].

Similar to other intracellular bacteria in in vitro and in vivo models [10,13,14], LF82 invasion process induces the synthesis of pro-inflammatory cytokines [8,10]. Of note, LF82 stimulates also the release of interferon-γ (IFN-γ) [2,15] which, in turn, increases both in vitro and in vivo the synthesis of CEACAM-6 [2,7,16,17]. In addition, IFN-γ has been shown to increase epithelial permeability by acting on tight junction proteins leading to intestinal barrier dysfunction [2].

In a recent study, it has been also demonstrated that LF82 is able to block the cellular cycle in S phase, inducing DNA damages in Caco-2 cells. In particular, the transcript levels of O6-methylguanine-DNA-methyltransferase (MGMT), a gene involved in the repair of DNA damages, have been found to decrease while the transcript levels of de novo methyltransferase (DNMT1), a gene involved in DNA methylation, have been found to increase when compared to the control [18]. These data led the authors to suggest that LF82 both blocks the cell DNA repair systems and increases the levels of DNA methylation, thus interfering with host gene expression [18].

Different therapeutic strategies targeting AIEC strains have been tested: strategies aimed at influencing bacterial adhesion and microbiota composition, direct to killing AIEC or to restore autophagy. Most of these are effective in controlling AIEC strains, and studies in human subjects are currently being planned [15,19,20,21,22].

Lactoferrin (Lf), a natural glycoprotein, has been demonstrated to exert anti-invasive activity against LF82 as well as to induce bacterial intracellular killing [10]. Lf is an 80 kDa multifunctional cationic glycoprotein of natural immunity expressed and secreted in humans by glandular epithelial cells and by neutrophils in infection and inflammation sites [23,24,25]. Bovine Lf (bLf), possessing high sequence homology and identical functions with human Lf, is applied in the majority of the in vitro and in vivo studies [24,25].

BLf exerts antimicrobial activity through its ability to chelate two ferric ions per molecule, limiting microbial growth at the infection sites [26]. On the other hand, by interacting with microbial surface, bLf exerts a microbicidal effect independent on iron-chelation [23].

Recently, Lf is emerging as an important natural glycoprotein able to elicit anti-inflammatory activity [25,26].

BLf also inhibits the bacterial adhesion to abiotic surfaces [27,28,29] or to host cells through its competitive binding with surface components of host cells and/or bacteria [10,30,31,32,33,34]. This glycoprotein also inhibits the entry into the host cells of Gram-negative and Gram-positive facultative or obligate intracellular bacteria [10,23,24,34,35,36,37]. Moreover, bLf plays a role in a variety of relevant biological activities modulating iron and inflammatory homeostasis [24,25,38,39,40,41]. As matter of fact, similarly to human Lf, bLf enters into the cell nucleus [42,43,44,45,46] where it binds to specific sequences of DNA, thus regulating gene transcription of anti-inflammatory cytokines [47]. Concerning bacterial intracellular survival, bLf, added to infected differentiated Caco-2 cells, has been found to partially inhibit AIEC LF82 intracellular growth [10]. Of note, even if the molecular mechanism through which bLf exerts its intracellular bactericidal activity remains unveiled, this function could be attributed to the direct interaction of bLf with bacteria or to an indirect action on cellular pathways, or to both. The bactericidal activity of IFN-γ has been also demonstrated in cell monolayers pre-incubated for 48 h with this cytokine before infection [10].

Here, we report that bLf, pre-incubated for 12 h with Caco-2 cells and removed before infection, reduces intracellular survival of LF82 and protects Caco-2 cells by bacterial-induced DNA damage.

## 2. Results

### 2.1. BLf and AIEC LF82 Adhesion, Invasion, and Survival into Caco-2 Cells

The ability of LF82 to adhere, invade and to survive into enterocytes, pre-treated with bLf or/and IFN-γ or untreated, was assessed.

Preliminary results showed that bLf pre-treatment did not interfere with the adhesion process at least at T0 (100 ± 29% vs. 98 ± 7%); the IFN-γ pre-treatment led to a significant increase in the percentage of adhesive bacteria (160 ± 17% vs. 98 ± 7%) with respect to untreated cells; in Caco-2 cells pre-treated with both, bLf was not able to contrast the IFN-γ action (150 ± 20% vs. 160 ± 17%).

**Figure 1:**
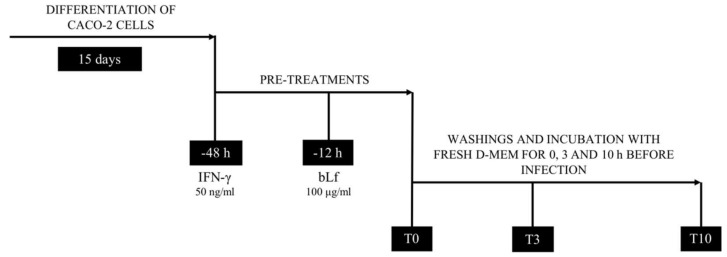
Experimental scheme for invasion and survival assays. Caco-2 cells were seeded at a density of 1 × 10^4^ cells/well in 24-well plates, incubated in DMEM plus 10% FBS at 37 °C in 5% CO_2_, and let to differentiate for 15 days. Differentiated Caco-2 cells were treated before infection with 50 ng/mL of interferon (IFN)-γ for 48 h or 100 µg/mL of bLf for 12 h or both. After the pre-treatments, differentiated Caco-2 cells were immediately infected with *E. coli* LF82 at a multiplicity of infection (MOI) of 1:10 at time 0 (T0) or after 3 and 10 h (T3 and T10).

The experimental scheme for invasion and survival assays is reported in Figure 1.

**Figure 2:**
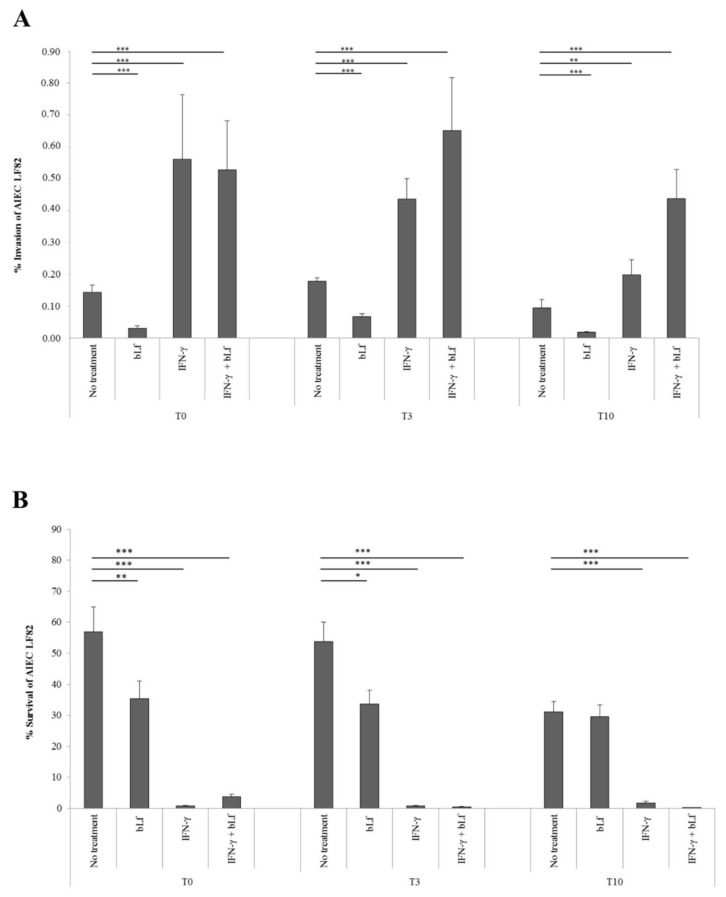
(**A**) Invasion efficiency of the adherent-invasive *E. coli* (AIEC) LF82 strain in differentiated Caco-2 cells pre-treated with bLf for 12 h or/and IFN-γ for 48 h. Pre-treated cells were infected immediately (T0) or after 3 (T3) or 10 h (T10) of incubation in fresh medium without IFN-γ and bLf. Invasion percentage values were calculated as the ratio between the numbers of intracellular bacteria and inoculum. Data represent the mean of five independent experiments in duplicate. **: p < 0.01; ***: p < 0.001 (paired Student’s t-test). (**B**) Survival efficiencies of AIEC LF82 strain in differentiated Caco-2 cells pre-treated with IFN-γ for 48 h and/or bLf for 12 h. Pre-treated cells were infected immediately (T0) or after 3 (T3) or 10 h (T10) of incubation in fresh medium without IFN-γ and bLf. Survival efficiency was calculated as the percentage of the ratio between intracellular bacteria recovered at 24 h and those recovered at 4 h. Data represent the mean of five independent experiments in duplicate. *: p < 0.05; **: p < 0.01; ***: p < 0.001 (paired Student’s t-test).

Regarding invasion, bLf pre-treatment for 12 h led to a significant decrease in the invasion efficiency of LF82 at T0 (0.03 ± 0.007% vs. 0.14 ± 0.023%), T3 (0.07 ± 0.009% vs. 0.18 ± 0.011%) and T10 (0.02 ± 0.002% vs. 0.10 ± 0.026%) with respect to untreated cells (Figure 2A).

Consistent with a previous paper [10], IFN-γ pre-treatment for 48 h led to a significant increase in the invasion rate of LF82 at T0 (0.56 ± 0.20% vs. 0.14 ± 0.023%), T3 (0.44 ± 0.063% vs. 0.18 ± 0.01%) and T10 (0.20 ± 0.047% vs. 0.10 ± 0.026%) as compared to untreated cells (Figure 2A). In cells pre-treated with both IFN-γ and bLf, LF82 invasion rates increased at all experimental times, similar to those observed in cells pre-treated with the sole IFN-γ at T0 (0.53 ± 0.15% vs. 0.56 ± 0.20%), T3 (0.65 ± 0.18% vs. 0.44 ± 0.063%) and T10 (0.44 ± 0.09% vs. 0.20 ± 0.047%) (Figure 2A).

Concerning the intracellular survival, compared to untreated cells, bLf pre-treatment significantly reduced the LF82 intracellular survival at T0 (35.41 ± 5.57% vs. 56.97 ± 8.05%) and T3 (33.64 ± 4.42% vs. 53.71 ± 6.40%), while it was ineffective at T10 (29.54 ± 3.86% vs. 31.10 ± 3.32%) (Figure 2B). IFN-γ pre-treatment significantly reduced the number of intracellular bacteria in all experimental conditions at T0 (0.80 ± 0.10% vs. 56.97 ± 8.05%), T3 (0.77 ± 0.22% vs. 53.71 ± 6.40%) and T10 (1.74 ± 0.68% vs. 31.10 ± 3.32%) with respect to untreated cells (Figure 2B). Finally, in the cells pre-treated with both IFN-γ and bLf, the LF82 intracellular survival was significantly reduced at T0 (3.68 ± 0.86% vs. 56.97 ± 8.05%), T3 (0.40 ± 0.13% vs. 53.71 ± 6.40%) and T10 (0.24 ± 0.09% vs. 31.10 ± 3.32%) with respect to that observed in untreated cells (Figure 2B).

### 2.2. BLf Varies Its Subcellular Localization over Time

Since bLf pre-treatment reduced LF82 invasion and survival at T0, T3 and T10, although to different extents, we performed a time course to investigate its putative presence and localization into the cells.

**Figure 3:**
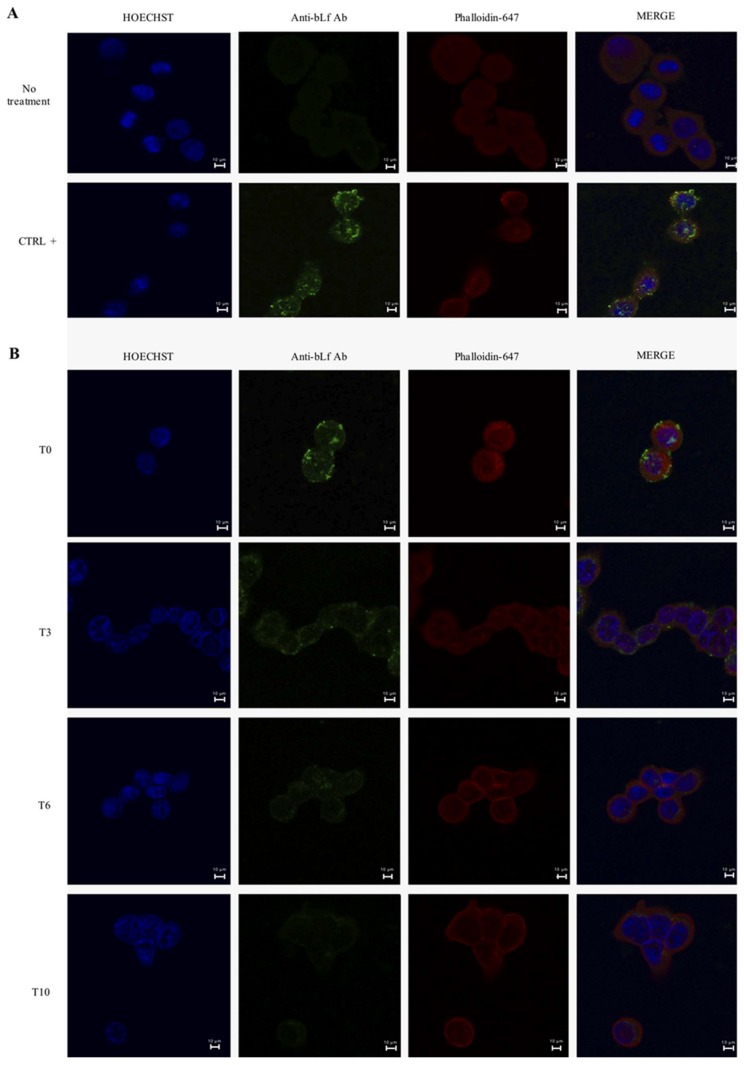

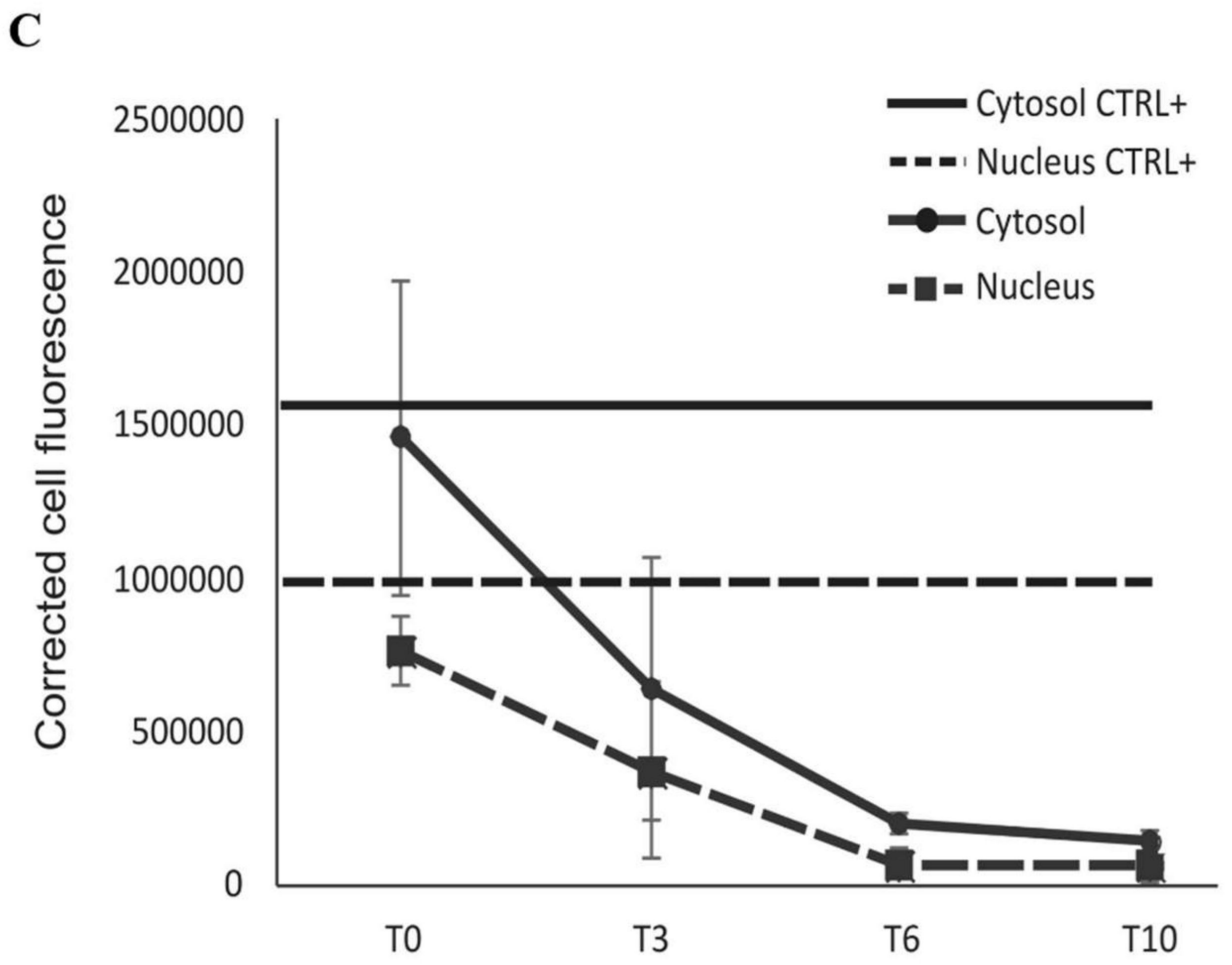
Time course of intracellular localization of bLf through confocal laser microscopy analysis. (**A**) As a positive control, the cells were incubated with culture medium containing bLf for 3 h. (**B**) In the pre-treated cells, images were acquired immediately after bLf removal (T0) and after 3 (T3), 6 (T6) and 10 (T10) of incubation in fresh medium without bLf. Scale bar: 10 μm. Caco-2 cells were stained with HOECHST (**blue**), phalloidin (**red**) and antibody against bLf (**green**). Images are representative of three independent experiments. (**C**) Quantification of bLf in cytosolic and nuclear compartments at different time-points. Values are expressed as mean ± SD relative to the analysis of ten different cells for each time-point. Levels of fluorescence relative to the positive control were also reported.

As a positive control for bLf nuclear localization, Caco-2 cells were incubated with bLf for 3 h [43,44,45,46]. As expected, after 3 h of incubation, bLf was found intracellularly, both into the nucleus and cytoplasm (Figure 3A). After 12 h of bLf pre-treatment, at T0, the protein was present into cells even if at weaker level with respect to the positive control and localized both into nucleus and cytoplasm (Figure 3B,C). At T3, the intensity of bLf signal decreased respect to T0 and the protein resulted still localized in both compartments (Figure 3B,C). At T6, bLf signal showed a relevant drop with a cytoplasmatic localization, while at T10, the protein signal was significantly reduced (Figure 3B,C).

### 2.3. BLf Does Not Influence CEACAM-6 Expression

To assess if the bLf capability to reduce LF82 invasion efficiency could be related to a modulation of the CEACAM-6 expression, Western blot analysis was performed. Indeed, it is well known that the invasion ability of AIEC strains is favored by CEACAM-6 receptor. The expression of this receptor, normally localized on the apical membrane of enterocytes, is increased by IFN-γ [2,9,15,48] and by LF82 itself [2,15,16,17].

**Figure 4:**
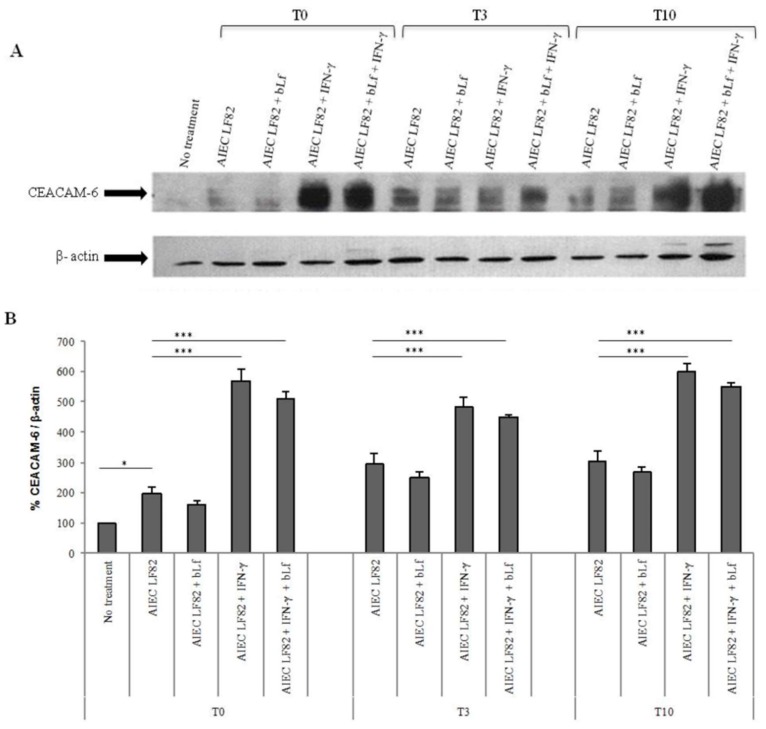
Effect of bLf pre-treatment on CEACAM-6 expression. (**A**) Western blot and (**B**) densitometric evaluations of CEACAM-6 expression in Caco-2 cells untreated or pre-treated with IFN-γ for 48 h and/or bLf for 12 h. Experiments were carried out immediately after treatment removal (T0) and after 3 (T3) and 10 h (T10) of incubation in fresh medium without IFN-γ and bLf. Densitometric evaluations of CEACAM-6 were normalized on β-actin expression. Data represent the mean of three independent experiments. *: *p* < 0.05; ***: *p* < 0.001 (Mann–Whitney U test).

Consequently, LF82 infection induced a significant increase of CEACAM-6 receptor with respect to uninfected cells (Figure 4A,B). When the cells pre-treated with IFN-γ were infected, a six-fold increase of CEACAM-6 receptor was observed at T0. Interestingly, a significantly higher expression of this receptor was maintained also at T3 and T10 (Figure 4A,B). In infected cells, the bLf pre-treatment induced a slight, although not significant, decrease of the CEACAM-6 receptor at all experimental times. Similar results were also obtained in Caco-2 cells pre-treated with both bLf and IFN-γ. In contrast to IFN-γ, in uninfected cells, bLf was not able to modify CEACAM-6 expression (data not shown).

### 2.4. BLf Does Not Induce Apoptosis in LF82-Infected Caco-2 Cells

Since bLf is able to modulate apoptosis [49,50,51,52], we evaluated whether the reduction of LF82 invasion and intracellular survival in bLf pre-treated cells could be due to bLf-induced apoptosis.

The number of viable cells in infected and uninfected cells at each time was evaluated by Trypan Blue staining. No difference in the number of viable cells was observed between infected and uninfected cells (data not shown).

**Figure 5:**
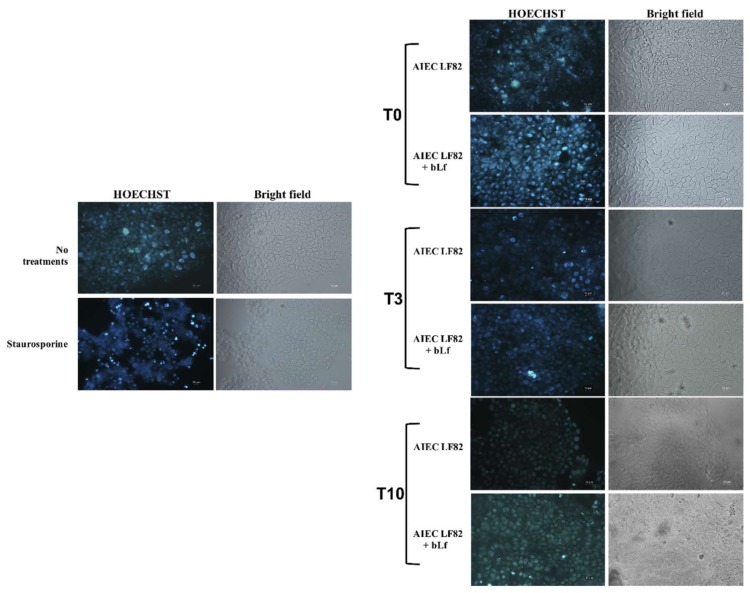
Effect of bLf pre-treatment on apoptosis of Caco-2 cells. Immunofluorescence and bright field microscopy were used to evaluate the apoptosis in Caco-2 cells pre-treated with bLf. The blue signal corresponds to HOECHST staining. Staurosporine was used as a positive control. Images were acquired immediately after bLf removal (T0) and after 3 (T3) and 10 h (T10) of incubation in fresh medium without bLf. Scale bar: 10 μm. Images are representative of three independent experiments.

Apoptosis, tested by HOECHST staining, showed, in contrast to staurosporine treatment, used as a positive control, that bLf pre-treatment did not lead to cellular apoptosis (Figure 5).

### 2.5. BLf Protects Caco-2 Cells from AIEC LF82-Induced DNA Damage

Recently, it has been demonstrated that AIEC strains induce DNA damage [18,53]. Therefore, we evaluated the bLf effect on LF82-induced DNA damage in Caco-2 cells through the Comet assay.

For this purpose, infected Caco-2 cells (MOI 1:10) were lysed, and the number of intracellular bacteria in all conditions was evaluated. The invasion efficiency was the same to that observed in invasion assays previously reported (Figure 2).

**Figure 6:**
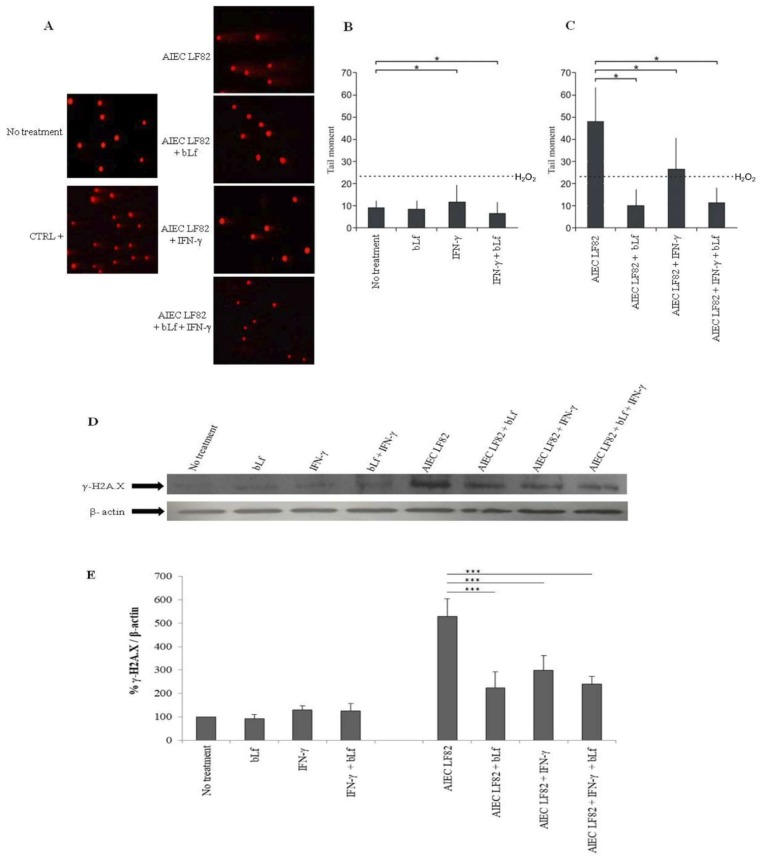
Effect of bLf pre-treatment on DNA damage induced by AIEC LF82. Alkaline single-cell gel electrophoresis (Comet) assay and Western blot were performed on Caco-2 cells untreated or pre-treated with IFN-γ for 48 h or/and bLf for 12 h and infected or not at T0. (**A**) Representative fluorescent microscopy images. Magnification 40x, scale bar: 10 μm. (**B,C**) Evaluation of DNA damage (for more details, see Materials and Methods). Dotted lines represent H_2_O_2_ quantification. Data represent the mean of three independent experiments in duplicate. Values are represented as mean ± SD. *: *p* ˂ 0.05 (Wilcoxon signed-rank test). (**D**) Representative Western blot of phosphorylated H2A.X (γ-H2A.X); (**E**) mean values of densitometric evaluation of γ-H2A.X expression normalized on β-actin. Data represent the mean of three independent experiments. ***: *p* ˂ 0.001 (Mann–Whitney U test).

In Comet assay, the results obtained in uninfected cells showed that bLf and/or IFN-γ pre-treatments did not induce significant comet formation compared to the positive control (H_2_O_2_ treated cells, broken line) (Figure 6B).

The highest values of DNA damage were observed in cells infected with LF82, thus confirming the previous observations [18,53] (Figure 6A,C). Of note, LF82 induced comet formation at much higher extent than H_2_O_2_ treated cells. Interestingly, bLf pre-treatment in infected cells revealed a strong protective action on DNA, as shown by the significant reduction of the comet formation (Figure 6A,C). Conversely, the sole IFN-γ pre-treatment, despite significantly reducing comet formation in infected cells, did not show an effective protective role against DNA damage, as demonstrated by the fact that the tail moments remained higher than those observed for H_2_O_2_-treated cells (Figure 6A,B). In infected cells pre-treated with both bLf and IFN-γ, the comet formation was strongly reduced, similar to that observed with the sole bLf (Figure 6A,C).

To deeply analyze the protective role of bLf against LF82-induced DNA damage, a Western blot on histone variant H2A.X was performed. As matter of fact, one of the earliest cell responses to DNA damage is the phosphorylation of the histone H2A.X (γ-H2A.X) [54].

The results showed that, in uninfected cells, both bLf and IFN-γ pre-treatments did not induce a significant induction of H2A.X phosphorylation compared to the control (Figure 6D,E). Conversely, LF82 strain induced a significant increase of histone γ-H2A.X compared to uninfected cells (Figure 6D,E).

In infected cells, the bLf pre-treatment significantly reduced the γ-H2A.X expression as well as IFN-γ pre-treatment did (Figure 6D,E). In cells pre-treated with both bLf and IFN-γ, the γ-H2A.X expression was significantly reduced, similar to that observed with bLf pre-treatment alone (Figure 6D,E).

## 3. Discussion

Some facultative and obligate intracellular Gram-positive and Gram-negative bacteria are capable not only of adhering, but also of entering into non-professional phagocytes, such as epithelial cells. Generally, bacterial adhesion/invasion of host cells is mediated by the binding of surface bacterial virulence determinants, such as adhesins and invasins, able to bind to a host integrin receptor thus playing a pivotal role in the entry process inside the host cells. Inside host cells, bacteria are in a protective niche in which they can replicate and persist, thus avoiding host defenses [55].

BLf is one of the most important natural glycoproteins and is able to reduce the bacterial adhesion/invasion efficiency in different cell models [10,23,24,30,56,57]. It appears that the binding of bLf to bacterial invasin or to cell glycosaminoglycans or heparan sulphates or to both can induce a decrease of bacterial–host cell interaction, thus inhibiting bacterial internalization [23,24,56,57].

In 2014, Frioni et al. [10] demonstrated that bLf is also able to kill intracellular LF82, a prototype of adherent-invasive *E. coli* (AIEC), able to adhere to, invade, survive and replicate into intestinal epithelial cells. LF82 infection and pro-inflammatory cytokine IFN-γ enhance either adhesion and invasion by up-regulating CEACAM-6, the main cell surface molecule for bacterial adhesion.

To try to unveil the mechanism(s) involved in these bLf activities against LF82, we examined the adhesive/invasive efficiency and the survival ability of this strain in cells pre-treated with bLf for 12 h and then removed before the infection. Furthermore, we pre-treated cells with IFN-γ, with or without bLf, to better simulate the in vivo conditions.

Here, we demonstrated for the first time that bLf pre-treatment of the Caco-2 cells is ineffective on bacterial adhesion, also in IFN-γ pre-treated cells, while it is able to decrease bacterial invasion up to T10 after its removal. One possible mechanism through which bLf reduces LF82 invasion could be related to an interaction between bLf and CEACAM-6 or to the modulation of CEACAM-6 receptor. As bLf does not interfere with adhesion process, is possible to hypothesize that bLf does not interact with CEACAM-6 and/or other structures involved in adhesion process. Furthermore, we showed that bLf did not significantly modulate the expression of this receptor in any experimental condition (Figure 4). Conversely, since IFN-γ is able to up-express CEACAM-6 receptor expression on Caco-2 cells (Figure 4) [2,9,48,58,59], the pre-treatment with IFN-γ, as expected, significantly increased the invasion efficiency up to T10 after its removal. When the cells were pre-treated with both, bLf was unable to contrast the IFN-γ effect on invasion efficiency. This could mean that, under inflammatory conditions, the bLf cannot protect the cells from AIEC infection. However, we could speculate that, independently from invasiveness, bLf and/or IFN-γ exert their effect thought intracellular killing, thus demonstrating a positive protective mechanism by these two molecules. As matter of fact, bLf pre-treatment significantly reduced the number of intracellular live bacteria. This effect was observed at both T0 and T3, while at T10, the number of intracellular bacteria was reduced, although at not significant level (Figure 2B).

Successively, we evaluated whether the reduction of LF82 invasion and intracellular survival by bLf could be due to an apoptotic process. The results have showed that bLf did not induce any apoptotic process in LF82-infected cells (Figure 5), nor the detachment of infected cells, as demonstrated by the viable cell counts performed at different times in all experimental conditions. These results led us to hypothesize that the LF82 killing could be related to intracellular bLf persistence (Figure 2B). For this purpose, the bLf subcellular localization, in all experimental conditions, was investigated. The results obtained showed that intracellular bLf content varied depending on the incubation time after its removal: at T0 it was present at higher concentration in both cytoplasm and nucleus; at T3 it was reduced in both the compartments; while at T10 it was significantly reduced, as shown by confocal microscopy (Figure 3). Interestingly, despite the protein signal being very low at T10, the bLf anti-invasive and anti-survival activity was still observed, even if at different extent. We could speculate that bLf could act by enhancing protective cellular pathways independent of the presence of bLf in the cells.

Recently, it has been demonstrated that AIEC strains were able to induce DNA damage, thus blocking the cell cycle in the S phase [18,60]. Therefore, a possible protective effect of bLf on AIEC-mediated genotoxicity was investigated. In this respect, Lf has been demonstrated to possess a remarkable antioxidant activity which, along with its ability to enter into the cell nucleus and bind to DNA [61], protects against DNA damage by directly scavenging hydroxyl radicals [62,63]. Indeed, Lf can exert its antioxidant activity by direct iron chelation or by regulating key antioxidant enzymes directly involved in reactive oxygen species (ROS) scavenging [64]. Moreover, recent studies have highlighted the ability of Lf to prevent DNA double-strand breaks, both in cell models challenged with aflatoxins [65] and in mice irradiated with sub-lethal X-rays [66].

Our results, obtained by Comet assay (Figure 6A,B,C) and Western blot analysis on the histone variant H2A.X (Figure 6D,E), clearly demonstrate the protective role of bLf against AIEC-induced DNA damage both in untreated and in IFN-γ-treated cells. At our knowledge, this is the first study showing that bLf can act as a potent protective molecule against bacterial-induced genotoxicity. Since this observation was done in cells in which bLf was localized in the nucleus (T0), it could be very interesting to evaluate the bLf activity also in bLf-treated cells in which this molecule is no longer found or found at very low level (T3 or T10, respectively).

However, how could the bLf-mediated DNA protection explain the decrease in AIEC invasion and survival within Caco-2 cells? Recently, Dalmasso G. et al. (2019) [67] showed that AIEC inhibits the autophagy response by manipulating host SUMOylation to replicate intracellularly. Moreover, the authors found that three hours of LF82 infection were enough to induce a profound decrease in both SUMO1- and SUMO2/3-conjugated protein levels, compared with uninfected cells. In addition, AIEC-induced protein deSUMOylation persisted until eight hours post-infection. Even if further experiments are needed, we could hypothesize that one of the possible mechanism(s) through which bLf exercises its protective role could be the prevention of the autophagy inhibition induced by AIEC infection.

In conclusion, the data obtained in this work show that bLf is able to reduce the invasion and survival of AIEC LF82 in human intestinal epithelial cells as well as to counteract bacterium-mediated genotoxicity, thus efficiently protecting the host cells. Although it will be necessary to perform further experiments using other AIEC strains to confirm the protective effect of bLf and to clarify the molecular mechanisms underneath, the results obtained may open a field of investigation regarding the putative use of this molecule in the therapeutic field. As matter of fact, several in vivo studies have already demonstrated that bLf administration is efficient in treating several pathologies due to infections caused by intracellular bacteria and inflammatory responses [25,34].

## 4. Materials and Methods

### 4.1. Lactoferrin

The purity of bLf (molecular weight of about 80 kDa) (Morinaga Milk Industries Co., Ltd., Tokyo, Japan) was checked by SDS-PAGE and silver nitrate staining. The bLf iron saturation was about 22%, as detected by optical spectroscopy at 468 nm based on an extinction coefficient of 0.54 (100% iron saturation, 1% solution). Before biological assays, bLf was sterilized by filtration (Millipore Corp., Bedford, MA, USA). The lipopolysaccharide (LPS) contamination of bLf, estimated by Limulus Amebocyte assay (LAL Pyrochrome kit, PBI International, Italy), was equal to 0.7 ± 0.06 ng/mg of bLf [68].

### 4.2. Bacterial Strain

*Escherichia coli* LF82, a prototype of an adherent-invasive *E. coli* (AIEC) strain isolated from a chronic ileal lesion of a patient with Crohn’s disease (CD), provided by Dr. Arlette Darfeuille-Michaud, Universitè d’Auvergne, Clermont-Ferrand, France, was used. To check purity, the strain was streaked on trypticase soy agar (TSA) plates (Oxoid LTD, Basingstoke, England) before the experiments. To prepare inoculum, AIEC LF82 strain was grown in brain heart infusion broth (BHI) (Oxoid LTD, Basingstoke, England) overnight at 37 °C. After the growth, AIEC LF82 was sub-cultured in fresh medium for 2 h at 37°C to obtain log-phase bacterial cultures to be used to infect Caco-2 cell monolayers.

### 4.3. Cell Culture

Human epithelial colorectal adenocarcinoma cell-line (Caco-2) was routinely maintained in Dulbecco’s modified Eagle’s medium (DMEM, Euroclone, Milan, Italy), supplemented with 1% penicillin/streptomycin and 10% fetal bovine serum (FBS, Gibco, New York, NY, USA) and maintained in a 5% CO_2_ atmosphere with 95% humidity at 37 °C. For differentiation, Caco-2 cells were seeded at a density of 1 × 10^4^ cells/well in 24-well plates, in the same culture medium, and let to differentiate for 15 days before infection.

### 4.4. Adhesion, Invasion and Survival Assays

The differentiated Caco-2 cells were untreated or treated before infection with 50 ng/mL of IFN-γ (Sigma-Aldrich, Milan, Italy) for 48 h [16] or 100 µg/mL of bLf for 12 h or with IFN-γ and bLf (Figure 1). In contrast to other reports, here, the bLf was added only 12 h before infection and was not present either during the infection period nor for the remaining 10 h. The 12 h of bLf pre-treatment was carried out in the absence of FBS to avoid the putative interference between bLf and serum transferrin. Therefore, longer times of pre-treatment led to cell stress, while shorter times did not show any variation of the intracellular bLf localization (see Section 4.6). Concerning IFN-γ, we decided to pre-treat cells with this cytokine to better simulate the in vivo conditions in order to have a positive control for invasion, intracellular bacteria survival, and CEACAM-6 expression. After the pre-treatment, differentiated Caco-2 cells were washed three times with phosphate buffered saline (PBS) in order to remove bLf or/and IFN-γ, and the wells were immediately infected with LF82 at a multiplicity of infection (MOI) of 1:10 at time 0 (T0) or after 3 and 10 h (T3 and T10) (Figure 1). The infection was carried out for 3 h at 37 °C in 5% CO_2_ atmosphere.

Regarding adhesion, the assay was carried out only at T0. After infection, Caco-2 cells washed three times with PBS were lysed in 0.1% (*v*/*v*) Triton X-100, and the number of adherent bacteria was determined by colony forming unit (CFU) counts on TSA plates. Bacterial adhesion was defined as the adhesion index, calculated as the percentage of adherent bacteria compared with the initial inoculum, taken as 100%.

For invasion assays, after infection at T0, T3 and T10, the supernatants were removed and Caco-2 cells were washed three times and newly incubated in fresh medium with 100 µg/mL gentamicin (Sigma-Aldrich, Milan, Italy) for 1 h to kill the extracellular bacteria. Caco-2 cells were lysed in 0.1% (*v*/*v*) Triton X-100 and plated on TSA plates to count intracellular CFUs. Invasion efficiency was calculated as the percentage of the ratio between intracellular bacteria and inoculum. Data represent the mean of five independent experiments in duplicate.

To evaluate intracellular killing, infected Caco-2 cells treated with 100 µg/mL gentamicin were incubated for a further 20 h at 37 °C in a 5% CO_2_ atmosphere in DMEM with a lower concentration of gentamicin (50 µg/mL). After a total of 24 h of incubation (T24), cells were lysed and plated on TSA plates as described above. The survival efficiency was calculated as the percentage of the ratio between intracellular bacteria recovered at 24 h and those recovered at 4 h. Data represent the mean of five independent experiments in duplicate.

The viability of Caco-2 cells was also determined by light microscopy after staining with 0.02% Trypan Blue (Sigma-Aldrich, Milan, Italy) and by cell counting at 4 h and at 24 h post-infection. Cell viability did not change significantly in all experimental conditions (data not shown).

### 4.5. Cell Apoptosis

Immunofluorescence analysis was performed on Caco-2 cells seeded at a density of 4 x 10^3^ cells/well in culture slides and left to differentiate for 15 days.

Cell apoptosis was evaluated on infected Caco-2 cells at T0, T3 and T10. After infection, cells were washed three times with PBS, fixed with 100% acetone (Carlo Erba, Milan, Italy) for 15 min, washed again, and permeabilized with Triton X-100 (0.2% in PBS). To stop the reaction, 5% bovine serum albumin (BSA) in PBS was added for 30 min, and the cells were stained with 1 µg/mL HOECHST 33342 for 5 min. Microscopy analysis was performed using a fluorescence microscope Leica DM5000 B. As a positive control, cells were incubated overnight with DMEM containing 2 μM of staurosporine, known to be an apoptotic inducer [69,70]. Furthermore, the number of viable cells in infected and uninfected cells at each time was evaluated by Trypan Blue staining.

### 4.6. Localization of Bovine Lactoferrin

To evaluate the presence and the localization of bLf, a time course was performed on bLf pre-treated uninfected cells. Briefly, Caco-2 cells were pre-treated with 100 µg/mL bLf for 12 h and washed three times. Then, cells were immediately fixed (T0) with 100% acetone for 15 min or successively fixed after bLf removal from culture medium and incubation at 37 °C in a 5% CO_2_ atmosphere with fresh DMEM for 3 (T3), 6 (T6), and 10 h (T10). After fixing, cells were treated with monoclonal mouse anti-bLf antibody (Santa Cruz Biotechnology, 1:100) for 1 h at 37 °C and then with polyclonal goat anti-mouse Alexa-Fluor 488 antibody (Invitrogen, 1:100) for 1 h. An incubation step with phalloidin-647 (Abcam, used according manufacturer’s instructions) was performed for 60 min. For the visualization of the nucleus, the cells were stained with 1 µg/mL HOECHST 33342 for 5 min.

As a positive control, cells were incubated with DMEM containing 100 µg/mL of bLf for 3 h [46]. After incubation, cells were washed, fixed, and labeled as detailed above.

Samples were examined by a confocal laser microscope (TCS SP8; Leica, Wetzlar, Germany) using a 40× 1.40–0.60 NA HCX Plan Apo oil BL objective at room temperature. Images are representative of three independent experiments.

The quantification of bLf was performed in ImageJ v 1.4.6 [71]. Briefly, the area relative to cytosol and the nucleus was selected by using the actin and DAPI signal, respectively. The integrated intensity relative to the green channel was determined, while the mean fluorescence of the background was calculated by sampling each image five times. The total bLf content of cytosol and nucleus was determined by calculating the corrected cell fluorescence (CCF) following the formula CCF = integrated density – (area of selected cell x mean fluorescence of background). At least ten different cells for each time-point were analyzed.

### 4.7. Comet Assay

AIEC LF82–related DNA damage in untreated or pre-treated Caco-2 cells was determined by neutral single-cell gel electrophoresis (SCGE). Briefly, differentiated Caco-2 cells, after pre-treatments and infection at T0, were washed and newly incubated in fresh medium with 100 µg/mL gentamicin (Sigma-Aldrich, Milan, Italy) for 1 h to kill the extracellular bacteria. Caco-2 cells were lysed in 0.1% (*v*/*v*) Triton X-100 and plated on TSA plates to count CFUs.

For the Comet assay, Caco-2 cells were trypsinized, and 20 µL of sample (~2 × 10^3^ cells/µL) for each experimental group was added to 180 µL of 0.6% low-melting-point agarose and spread on glass slides. The agarose-suspended cells were covered with a coverslip and left at 4 °C for 2–3 min to solidify. After agarose solidification, the coverslips were removed and the slides were immersed overnight in the lysis solution (2 M NaCl, 30 mM EDTA, 10 mM Tris-HCl buffer pH 10, 1% *v*/*v* Triton X-100) at 4 °C.

The slides were rinsed three times with Tris/Borate/Ethylenediaminetetraacetic acid (EDTA) (TBE) electrophoresis buffer, kept at 4 °C in order to remove detergent, and subsequently transferred into a horizontal electrophoresis tank filled with buffer. The electrophoretic run was maintained for 25–30 min at 0.6 V/cm. Slides were stained with 2–5 µM ethidium bromide (EtBr) for 15–20 min. Caco-2 cells treated with 20 µM H_2_O_2_ for 20 min at 4 °C were used as a positive control. A total of 50 randomly captured comets for each slide were randomly examined at 40× magnification using a fluorescence microscope (Leica DM5000 B, Wetzlar, Germany).

Images were acquired by using a digital image acquisition system, and the integrated intensity profile for each cell was computed. Undamaged cells presented an intact nucleus, while damaged ones showed a “migrated tail” formed by the differential migration of fragments produced by DNA breakdown. In order to evaluate the DNA damage, the comet tail moment was calculated by using CASP V1.2.2 software [72]. The comet tail moment is positively correlated with the level of DNA breakage in a cell and is calculated as the product of the tail length (DNA migration) and tail intensity (% of present DNA in the tail estimated by the fraction of DNA in the comet tail).

The mean value of the tail moment in a particular sample was taken as an index of DNA damage in this sample. Data represent the mean of three independent experiments in duplicate.

### 4.8. Western Blot Analysis

Western blot was performed to evaluate CEACAM-6 and γ-H2A.X expression. For this purpose, differentiated Caco-2 cells, after pre-treatments and infection at T0, were scraped in 1 mL of PBS containing 1 mM of phenylmethylsulfonyl fluoride (PMSF), harvested by centrifugation at 2500× *g* for 5 min, and stored at −80 °C. Cells were lysed in 300 µL 3-(*N*-morpholino)propanesulfonic acid (MOPS), 25 mM pH 7.4/NaCl 150 mM/Triton 1% containing 1 mM of PMSF, Leupeptin, and Pepstatin 2 µM in ice for 1 h. The total protein content of samples was measured by the Bradford assay. For SDS-PAGE, 20 µg of total protein was loaded per lane, SDS sample buffer containing DTT was added, and samples were loaded after heat treatment. Primary antibodies were mouse monoclonal anti-CEACAM-6 (Santa Cruz Biotechnology, Santa Cruz, CA, USA) (1:10,000), mouse monoclonal anti-γ-H2A.X (Santa Cruz Biotechnology, Santa Cruz, CA, USA) (1:5,000) and mouse monoclonal anti-β-actin (Sigma-Aldrich, Milan, Italy) (1:10,000). After incubation with the appropriate secondary HRP-conjugated antibody, blots were developed with ECL Prime (GE Healthcare, Milan, Italy). CEACAM-6 and γ-H2A.X levels were normalized on β-actin by densitometry analysis, performed with ImageJ software. Data represent the mean of three independent experiments.

### 4.9. Statistical Analysis

Results are expressed as means ± standard deviations (SD). The paired Student’s t-test was used to determine the presence of statistically significant differences between treatments for invasion and survival efficiency, while the Wilcoxon signed-rank test was used for tail moment. For Western blots, the Mann–Whitney U test was used. All statistical analysis analyzes were performed by using Prism v7 software (GraphPad Software, USA). In all cases, a p-value less than or equal to 0.05 was considered statistically significant.

## References

[1] Darfeuille-Michaud A., Boudeau J., Bulois P., Neut C., Glasser A., Barnich N., Colombel J.F. (2004). Hight prevalence of adherent-invasive *Escherichia coli* associated with ileal mucosa in Crohn’s disease. Gastroenterology.

[2] Shawki A., McCole D.F. (2017). Mechanisms of intestinal epithelial barrier dysfunction by adherent-invasive *Escherichia coli*. Cell. Mol. Gastroenterol. Hepatol..

[3] Rolhion N., Darfeuille-Michaud A. (2007). Adherent-Invasive *Escherichia coli* in Inflammatory Bowel Disease. Inflamm. Bowel Dis..

[4] Sasaki M., Sitaraman S.V., Babbin B.A., Gerner-Smidt P., Ribot E.M., Garrett N., Alpern J.A., Akyildiz A., Theiss A.L., Nusrat A. (2007). Invasive *Escherichia coli* are a feature of Crohn’s disease. Lab. Invest..

[5] Martinez-Medina M., Aldeguer X., Lopez-Siles M., Gonzalez-Huix F., Lopez-Oliu C., Dahbi G., Blanco J.E., Garcia-Gil L.J., Darfeuille-Michaud A. (2009). Molecular diversity of *Escherichia coli* in the human gut: New ecological evidence supporting the role of adherent-invasive *E. coli* (AIEC) in Crohn’s disease. Inflamm. Bowel Dis..

[6] Chassaing B., Etienne-Mesmin L., Bonnet R., Darfeuille-Michaud A. (2013). Bile salts induce long polar fimbriae expression favouring Crohn’s disease-associated adherent-invasive *Escherichia coli* interaction with Peyer’s patches. Environ. Microbiol..

[7] Barnich N., Carvalho F.A., Glasser A.L., Darcha C., Jantscheff P., Allez M., Peeters H., Bommelaer G., Desreumaux P., Colombel J.F. (2007). CEACAM6 acts as a receptor for adherent-invasive *E. coli*, supporting ileal mucosa colonization in Crohn disease. J. Clin. Invest..

[8] Carvalho F.A., Barnich N., Sivignon A., Darcha C., Chan C.H., Stanners C.P., Darfeuille-Michaud A. (2009). Crohn’s disease adherent-invasive *Escherichia coli* colonize and induce strong gut inflammation in transgenic mice expressing human CEACAM. J. Exp. Med..

[9] Barnich N., Darfeuille-Michaud A. (2007). Adherent-invasive *Escherichia coli* and Crohn’s disease. Curr. Opin. Gastroenterol..

[10] Frioni A., Conte M.P., Cutone A., Longhi C., Musci G., di Patti M.C., Natalizi T., Marazzato M., Lepanto M.S., Puddu P. (2014). Lactoferrin differently modulates the inflammatory response in epithelial models mimicking human inflammatory and infectious diseases. BioMetals.

[11] Glasser A.L., Boudeau J., Barnich N., Perruchot M.H., Colombel J.F., Darfeuille-Michaud A. (2001). Adherent invasive *Escherichia coli* strains from patients with Crohn’s disease survive and replicate within macrophages without inducing host cell death. Infect. Imm..

[12] Rahman K., Sasaki M., Nusrat A., Klapproth J.M. (2014). Crohn’s disease-associated *Escherichia coli* survive in macrophages by suppressing NFκB signaling. Inflamm. Bowel Dis..

[13] Berlutti F., Schippa S., Morea C., Sarli S., Perfetto B., Donnarumma G., Valenti P. (2006). Lactoferrin down regulates pro-inflammatory cytokines up-expressed in intestinal epithelial cells infected with invasive or noninvasive *Escherichia coli* strains. Biochem. Cell Biol..

[14] Valenti P., Frioni A., Rossi A., Ranucci S., De Fino I., Cutone A., Rosa L., Bragonzi A., Berlutti F. (2017). Aerosolized bovine lactoferrin reduces neutrophils and pro-inflammatory cytokines in mouse models of *Pseudomonas aeruginosa* lung infections. Biochem. Cell Biol..

[15] Palmela C., Chevarin C., Xu Z., Torres J., Sevrin G., Hirten R., Barnich N., Ng S.C., Colombel J.F. (2018). Adherent-invasive *Escherichia coli* in inflammatory bowel disease. Gut.

[16] Rolhion N., Barnich N., Bringer M.A., Glasser A.L., Ranc J., Hébuterne X., Hofman P., Darfeuille-Michaud A. (2010). Abnormally expressed ER stress response chaperone Gp96 in CD favours adherent-invasive *Escherichia coli* invasion. Gut.

[17] Fahlgren A., Baranov V., Frängsmyr L., Zoubir F., Hammarström M.L., Hammarström S. (2003). Interferon-gamma tempers the expression of carcinoembryonic antigen family molecules in human colon cells: A possible role in innate mucosal defence. Scand. J. Immunol..

[18] Mazzarella G., Perna A., Marano A., Lucariello A., Rotondi Aufieri V., Melina R., Melina R., Guerra G., Taccone F.S., Iaquinto G. (2017). Pathogenic Role of Associated Adherent-Invasive *Escherichia Coli* in Crohn’s disease. J. Cell. Physiol..

[19] Brument S., Sivignon A., Dumych T.I., Moreau N., Roos G., Guérardel Y., Chalopin T., Deniaud D., Bilyy R.O., Darfeuille-Michaud A. (2013). Thiazolylaminomannosides as potent antiadhesives of type 1 piliated *Escherichia coli* isolated from Crohn’s disease patients. J. Med. Chem..

[20] Sivignon A., Yan X., Alvarez Dorta D., Bonnet R., Bouckaert J., Fleury E., Bernard J., Gouin S.G., Darfeuille-Michaud A., Barnich N. (2015). Development of heptylmannoside-based glycoconjugate antiadhesive compounds against Adherent-Invasive *Escherichia coli* bacteria associated with Crohn’s disease. MBio.

[21] Alvarez Dorta D., Sivignon A., Chalopin T., Dumych T.I., Roos G., Bilyy R.O., Deniaud D., Krammer E.M., de Ruyck J., Lensink M.F. (2016). The Antiadhesive Strategy in Crohn’s Disease: Orally Active Mannosides to Decolonize Pathogenic *Escherichia coli* from the Gut. ChemBioChem.

[22] Chalopin T., Alvarez Dorta D., Sivignon A., Caudan M., Dumych T.I., Bilyy R.O., Deniaud D., Gouin S.G. (2016). Second generation of thiazolylmannosides, FimH antagonists for *E. coli*-induced Crohn’s disease. Org. Biomol. Chem..

[23] Valenti P., Antonini G. (2005). Lactoferrin: An important host defence against microbial and viral attack. Cell. Mol. Life Sci..

[24] Rosa L., Cutone A., Lepanto M.S., Paesano R., Valenti P. (2017). Lactoferrin: A Natural Glycoprotein Involved in Iron and Inflammatory Homeostasis. Int. J. Mol. Sci..

[25] Lepanto M.S., Rosa L., Paesano R., Valenti P., Cutone A. (2019). Lactoferrin in Aseptic and Septic Inflammation. Molecules.

[26] Ward P.P., Paz E., Conneely O.M. (2005). Multifunctional roles of lactoferrin: A critical overview. Cell. Mol. Life Sci..

[27] Visca P., Berlutti F., Vittorioso P., Dalmastri C., Thaller M.C., Valenti P. (1989). Growth and adsorption of *Streptococcus mutans* 6715-13 to hydroxyapatite in the presence of lactoferrin. Med. Microbiol. Immunol..

[28] Hirano Y., Tamura M., Hayashi K. (2000). Inhibitory effect of lactoferrin on the adhesion of *Prevotella nigrescens* ATCC 25261 to hydroxyapatite. J. Oral Sci..

[29] Williams T.J., Schneider R.P., Willcox M.D. (2003). The effect of protein-coated contact lenses on the adhesion and viability of Gram negative bacteria. Curr. Eye Res..

[30] Longhi C., Conte M.P., Seganti L., Polidoro M., Alfsen A., Valenti P. (1993). Influence of lactoferrin on the entry process of *Escherichia coli* HB101 (pRI203) in HeLa cells. Med. Microbiol. Immunol..

[31] Alugupalli K.R., Kalfas S. (1997). Characterization of the lactoferrin-dependent inhibition of the adhesion of *Actinobacillus actinomycetemcomitans*, *Prevotella intermedia* and *Prevotella nigrescens* to fibroblasts and to a reconstituted basement membrane. APMIS.

[32] Kawasaki Y., Tazume S., Shimizu K., Matsuzawa H., Dosako S., Isoda H., Tsukiji M., Fujimura R., Muranaka Y., Isihida H. (2000). Inhibitory effects of bovine lactoferrin on the adherence of enterotoxigenic *Escherichia coli* to host cells. Biosci. Biotechnol. Biochem..

[33] Berlutti F., Morea C., Battistoni A., Sarli S., Cipriani P., Superti F., Amendolia M.G., Valenti P. (2005). Iron availability influences aggregation, biofilm, adhesion and invasion of *Pseudomonas aeruginosa* and *Burkholderia cenocepacia*. Int. J. Immunopathol. Pharmacol..

[34] Sessa R., Di Pietro M., Filardo S., Bressan A., Rosa L., Cutone A., Frioni A., Berlutti F., Paesano R., Valenti P. (2017). Effect of bovine lactoferrin on *Chlamydia trachomatis* infection and inflammation. Biochem. Cell Biol..

[35] Antonini G., Catania M.R., Greco R., Longhi C., Pisciotta M.G., Seganti L., Valenti P. (1997). Anti-invasive activity of bovine lactoferrin towards *Listeria monocytogenes*. J. Food Prot..

[36] Ajello M., Greco R., Giansanti F., Massucci M.T., Antonini G., Valenti P. (2002). Anti-invasive activity of bovine lactoferrin towards group A streptococci. Biochem. Cell Biol..

[37] Willer Eda M., Lima Rde L., Giugliano L.G. (2004). In vitro adhesion and invasion inhibition of *Shigella dysenteriae*, *Shigella flexneri* and *Shigella sonnei* clinical strains by human milk proteins. BMC Microbiol..

[38] Cutone A., Rosa L., Lepanto M.S., Scotti M.J., Berlutti F., Bonaccorsi di Patti M.C., Musci G., Valenti P. (2017). Lactoferrin efficiently counteracts the inflammation-induced changes of the iron homeostasis system in macrophages. Front. Immunol..

[39] Bonaccorsi di Patti M.C., Cutone A., Polticelli F., Rosa L., Lepanto M.S., Valenti P., Musci G. (2018). The ferroportin-ceruloplasmin system and the mammalian iron homeostasis machine: Regulatory pathways and the role of lactoferrin. BioMetals.

[40] Lepanto M.S., Rosa L., Cutone A., Conte M.P., Paesano R., Valenti P. (2018). Efficacy of Lactoferrin Oral Administration in the Treatment of Anemia and Anemia of Inflammation in Pregnant and Non-pregnant Women: An Interventional Study. Front. Immunol..

[41] Cutone A., Lepanto M.S., Rosa L., Scotti M.J., Rossi A., Ranucci S., De Fino I., Bragonzi A., Valenti P., Musci G. (2019). Aerosolized Bovine Lactoferrin Counteracts Infection, Inflammation and Iron Dysbalance in A Cystic Fibrosis Mouse Model of *Pseudomonas aeruginosa* Chronic Lung Infection. Int. J. Mol. Sci..

[42] Penco S., Scarfi S., Giovine M., Damonte G., Millo E., Villaggio B., Passalacqua M., Pozzolini M., Garrè C., Benatti U. (2001). Identification of an import signal for, and the nuclear localization of, human lactoferrin. Biotechnol. Appl. Biochem..

[43] Ashida K., Sasaki H., Sasaki Y.A., Loönnerdal B. (2004). Cellular internalization of lactoferrin in intestinal epithelial cells. BioMetals.

[44] Suzuki Y.A., Lopez V., Lönnerdal B. (2005). Mammalian lactoferrin receptors: Structure and function. Cell. Mol. Life Sci..

[45] Suzuki Y.A., Wong H., Ashida K.Y., Schryvers A.B., Loönnerdal B. (2008). The N1 domain of human lactoferrin is required for internalization by caco-2 cells and targeting to the nucleus. Biochemistry.

[46] Paesano R., Natalizi T., Berlutti F., Valenti P. (2012). Body iron delocalization: The serious drawback in iron disorders in both developing and developed countries. Pathog. Glob. Health.

[47] Kim C.W., Lee T.H., Park K.H., Choi S.Y., Kim J. (2012). Human lactoferrin suppresses TNF-α-induced intercellular adhesion molecule-1 expression via competition with NF-kB in endothelial cells. FEBS Lett..

[48] Barnich N., Darfeuille-Michaud A. (2010). Abnormal CEACAM6 expression in Crohn disease patients favors gut colonization and inflammation by adherent-invasive *E. coli*. Virulence.

[49] Valenti P., Greco R., Pitari G., Rossi P., Ajello M., Melino G., Antonini G. (1999). Apoptosis of Caco-2 intestinal cells invaded by *Listeria monocytogenes*: Protective effect of lactoferrin. Exp. Cell Res..

[50] Longhi C., Conte M.P., Ranaldi S., Penta M., Valenti P., Tinari A., Superti F., Seganti L. (2005). Apoptotic death of *Listeria monocytogenes*-infected human macrophages induced by lactoferricin B, a bovine lactoferrin-derived peptide. Int. J. Immunopathol. Pharmacol..

[51] Blais A., Fan C., Voisin T., Aattouri N., Dubarry M., Blachier F., Tome D. (2014). Effects of lactoferrin on intestinal epithelial cell growth and differentiation: An in vivo and in vitro study. BioMetals.

[52] Nguyen D.N., Jiang P., Stensballe A., Bendixen E., Sangild P.T., Chatterton D.E. (2016). Bovine lactoferrin regulates cell survival, apoptosis and inflammation in intestinal epithelial cells and preterm pig intestine. J. Proteomics.

[53] Tyrer P.C., Frizelle F.A., Keenan J.I. (2014). *Escherichia coli*-derived outer membrane vesicles are genotoxic to human enterocyte-like cells. Infect. Agents Cancer.

[54] Kuefner M.A., Brand M., Engert C., Schwab S.A., Uder M. (2015). Radiation Induced DNA Double-Strand Breaks in Radiology. RoFo.

[55] Thakur A., Mikkelsen H., Jungersen G. (2019). Intracellular Pathogens: Host Immunity and Microbial Persistence Strategies. J. Immunol. Res..

[56] Sessa R., Di Pietro M., Filardo S., Bressan A., Mastromarino P., Biasucci A.V., Rosa L., Cutone A., Berlutti F., Paesano R. (2017). Lactobacilli-lactoferrin interplay in *Chlamydia trachomatis* infection. Pathog. Dis..

[57] Valenti P., Rosa L., Capobianco D., Lepanto M.S., Schiavi E., Cutone A., Paesano R., Mastromarino P. (2018). Role of lactobacilli and lactoferrin in the mucosal cervicovaginal defense. Front. Immunol..

[58] Bretin A., Lucas C., Larabi A., Dalmasso G., Billard E., Barnich N., Bonnet R., Nguyen H.T.T. (2018). AIEC infection triggers modification of gut microbiota composition in genetically predisposed mice, contributing to intestinal inflammation. Sci. Rep..

[59] Lashermes A., Boudieu L., Barbier J., Sion B., Gelot A., Barnich N., Ardid D., Carvalho F.A. (2018). Adherent-Invasive *E. coli* enhances colonic hypersensitivity and P2X receptors expression during post-infectious period. Gut Microbes..

[60] Cañas M.A., Giménez R., Fábrega M.J., Toloza L., Baldomà L., Badia J. (2016). Outer Membrane Vesicles from the Probiotic *Escherichia coli* Nissle 1917 and the Commensal ECOR12 Enter Intestinal Epithelial Cells via Clathrin-Dependent Endocytosis and Elicit Differential Effects on DNA Damage. PLoS ONE.

[61] Bennett R.M., Davis J. (1982). Lactoferrin interacts with deoxyribonucleic acid: A preferential reactivity with double-stranded DNA and dissociation of DNA-anti-DNA complexes. J. Lab. Clin. Med..

[62] Kruzel M.L., Actor J.K., Radak Z., Bacsi A., Saavedra-Molina A., Boldogh I. (2010). Lactoferrin decreases LPS-induced mitochondrial dysfunction in cultured cells and in animal endotoxemia model. Innate Immun..

[63] Ogasawara Y., Imase M., Oda H., Wakabayashi H., Ishii K. (2014). Lactoferrin directly scavenges hydroxyl radicals and undergoes oxidative self-degradation: A possible role in protection against oxidative DNA damage. Int. J. Mol. Sci..

[64] Kruzel M.L., Zimecki M., Actor J.K. (2017). Lactoferrin in a Context of Inflammation-Induced Pathology. Front. Immunol..

[65] Zheng N., Zhang H., Li S., Wang J., Liu J., Ren H., Gao Y. (2018). Lactoferrin inhibits aflatoxin B1- and aflatoxin M1-induced cytotoxicity and DNA damage in Caco-2, HEK, Hep-G2, and SK-N-SH cells. Toxicon.

[66] Feng L., Li J., Qin L., Guo D., Ding H., Deng D. (2018). Radioprotective effect of lactoferrin in mice exposed to sublethal X-ray irradiation. Exp. Ther. Med..

[67] Dalmasso G., Nguyen H.T.T., Faïs T., Massier S., Barnich N., Delmas J., Bonnet R. (2019). Crohn’s Disease-Associated Adherent-Invasive Escherichia coli Manipulate Host Autophagy by Impairing SUMOylation. Cells.

[68] Rosa L., Cutone A., Lepanto M.S., Scotti M.J., Conte M.P., Paesano R., Valenti P. (2018). Physico-chemical properties influence the functions and efficacy of commercial bovine lactoferrins. BioMetals.

[69] Labadie K., Saulnier A., Martin-Latil S., Colbère-Garapin F. (2007). Reduced apoptosis in human intestinal cells cured of persistent poliovirus infection. J. Virol..

[70] Eidet J.R., Pasovic L., Maria R., Jackson C.J., Utheim T.P. (2014). Objective assessment of changes in nuclear morphology and cell distribution following induction of apoptosis. Diagn. Pathol..

[71] Schneider C.A., Rasband W.S., Eliceiri K.W. (2012). NIH Image to ImageJ: 25 years of image analysis. Nat. Methods.

[72] Końca K., Lankoff A., Banasik A., Lisowska H., Kuszewski T., Góźdź S., Koza Z., Wojcik A. (2003). A cross platform public domain PC image analysis program for the comet assay. Mutat. Res..

